# Authors’ Responses to Peer Review Reports for "Angiotensin Converting Enzyme 1 Expression in the Leukocytes of Adults Aged 64 to 67 Years"

**DOI:** 10.2196/45280

**Published:** 2023-01-20

**Authors:** Valquiria Bueno, Pedro Henrique Destro, Daniela Teixeira, Daniela Frasca

**Affiliations:** 1 Division of Immunology Department of Microbiology Immunology and Parasitology Federal University of São Paulo São Paulo Brazil; 2 Department of Microbiology and Immunology Miller School of Medicine University of Miami Miami, FL United States

**Keywords:** aging, angiotensin converting enzyme, lymphocytes, immunosenescence, inflammaging

*This is the authors’ response to peer-review reports for “Angiotensin Converting Enzyme 1 Expression in the Leukocytes of Adults Aged 64 to 67 Years”* [1].

## Round 1 Review

### Reviewer Heikki Vapaatalo [2]

Dear reviewer, thank you very much for the insightful suggestions; the manuscript improved a lot with the suggested changes. Please find our point-by-point answers to the raised questions. In the main text, all changes are highlighted in yellow. I hope that with the changes made, the new version is suitable for publication.

#### General Comments

1. The study is interesting, and the title promises for me more than the manuscript finally contains.

Answer: The manuscript is part of a project aiming to study ACE1 and ACE2 expression in cells from the immune system of aging and young adults. These initial results suggest that ACE1 (and probably ACE2) somehow plays a role in the process of aging.

2. The background, question, and the aim are relevant as explained in the Introduction.

Answer: We included some information in the Introduction, trying to link ACE1 expression in tissue cells and age-related diseases, as follows:

“ACE1 has been suggested to influence age-related diseases (ie, Alzheimer disease, sarcopenia, and cancer) but the associated mechanisms are still under investigation. *ACE1* polymorphisms have been correlated with susceptibility to Alzheimer disease [15,16]. In addition, it was shown recently that in normal aging, ACE1 expression is increased in brain homogenates, and this expression is unchanged in early stages of Alzheimer disease [17]. Regarding sarcopenia, Yoshihara et al [18] found a weak correlation between *ACE1* polymorphism and physical function. In cancer (gastric or colorectal), patients presented higher expression of ACE1 in tumors than in healthy tissues [19,20].”

3. The major concerns the small size of the material (6 subjects), the small age difference (64-67 years), and the lack of younger controls.

Answer: We agree that the small number of studied subjects is a limitation of this study. In spite of the interesting results suggesting that ACE1 expression could be linked to the health status, it was not possible to perform correlation analysis due to the small sample size. Even though there is a small chronological difference among the subjects, the biological aging is very different among them and reflects the genetics, lifestyle, nutrition, and comorbidities. Another limitation is the lack of younger controls to compare with the subjects studied. Our next steps are to include younger controls, to increase the number of studied subjects, and, if possible, to obtain samples from older subjects (ie, aged 70-80, 80, and >80 years).

#### Specific Comments

1. Title: ACE seems better than ACE1; or, does the sophisticated, elegant method include both ACEs?

Answer: We evaluated only ACE1 expression, and thus, the title, abstract, and main text were changed to indicate ACE1 instead of ACE. We decided to change the title to “Angiotensin Converting Enzyme 1 Expression in the Leukocytes of Adults Aged 64 to 67 Years.”

2. Introduction: in the last chapter, the author should explain in more detail how Pawelec et al [3], Alves et al [4], Alves and Bueno [5], and Bueno et al [6] suggest that “ACE1 plays an important role in the aging process.” Does “ACE1 plays” mean, that ACE1 is somehow regulating the aging process or are ACE1 levels changed with age?

Answer: These cited studies show that age-related diseases occurring in older adults are associated with changes in the immune system. To complete the text, we added the following:

“ACE1 has been suggested to influence age-related diseases (ie, Alzheimer disease, sarcopenia, and cancer) but the associated mechanisms are still under investigation. *ACE1* polymorphisms have been correlated with susceptibility to Alzheimer disease [15,16]. In addition, it was shown recently that in normal aging, ACE1 expression is increased in brain homogenates, and this expression is unchanged in early stages of Alzheimer disease [17]. Regarding sarcopenia, Yoshihara et al [18] found a weak correlation between *ACE1* polymorphism and physical function. In cancer (gastric or colorectal), patients presented higher expression of ACE1 in tumors than in healthy tissues [19,20].”

Methods:

1. The N value of the subjects should be mentioned here, as well the relation of females and males.

Answer: Text was corrected as suggested: “Blood was collected from adults (n=6, four females and 2 males) aged 64-67 years in 2015.”

2. Do the authors really regard 64-67 years “older age” nowadays?

Answer: Nowadays, the most common term used for individuals older than 65 years is “older adults.”

3. Why were the initial assays done many years after the collection of blood samples? Are the samples still useable and not destroyed?

Answer: Samples are part of UNIFESP Biobank and have been maintained in adequate conditions. We wanted to test cells from a period before the COVID-19 pandemic and those samples were the only ones that served our purpose. We compared samples used in this study with fresh blood samples (cell viability and percentage of CD4^+^, CD8^+^, and CD19^+^ cells) and the results showed good preservation of the cells.

4. Did the subjects have some diseases or were taking drugs because they possibly were from a hospital sample bank?

Answer: The samples are part of UNIFESP Biobank, but unfortunately, we do not have information about diseases and medicaments.

5.Provide the companies’ details.

Answer: Changes were made as required: “ACE CD143 fluorescein isothiocyanate (R&D Systems).”

Results:

1. “Table 1 shows that older adults…..” The comparison between the present data and historical studies belongs to the Discussion.

Answer: Changes were made as required.

2. Also, provide individual ages and genders of the subjects in Table 1.

Answer: The manuscript version sent to medrxiv@medrxiv.org had age and gender on tables, but due to their request, any possible variable that could identify the study participant had to be removed. Hence, the present version these variables are not shown.

3.What do *P* values mean here—what is being compared, or are interindividual differences being highlighted in the particular variables? This should be explained.

Answer: We used *P* values for interindividual differences in each variable, since individuals age differently (biological aging); thus, physiological parameters could be affected by genetics, lifestyle, nutrition, and comorbidities. It is now explained in the Methods section.

4. The numbering of tables and the text seems confusing to me. Only 3 tables, but in the text, 4 are mentioned. Table 4 does not exist.

Answer: For some reason, Table 2 is missing in the main text. Please find the new version with Table 2 included.

5. It would be good to have a list of abbreviations used in the description of the cell types for an unfamiliar reader.

Answer: In each figure and table, we are now providing a description of cells evaluated.

Discussion:

1. A major part of the discussion deals with previous publications and not meaning or clinical significance of the present findings and comparison between the present and earlier studies.

Answer: The discussion was changed as suggested:

“Our results show that for the studied population, chronological aging and biological aging are asynchronous. Even among individuals with a small chronological difference (64 to 67 years), there is heterogeneity in physiological parameters such as glucose, urea, glycated hemoglobin, and CRP. Changes in the same functional parameters have been reported by Carlsson et al [22] and Helmerson-Karlqvist [23] in healthy older adults. Carlsson’s [22] study found that the CRP level was 2.6% with a coefficient variation of 1.4%, whereas in our study, we observed higher levels of CRP in 5 out of 6 individuals. Increased CRP levels have been associated with inflammaging, and our findings show that the study population has changes in functional parameters, which are likely associated with an inflammatory profile [24].

The link between the RAS and inflammation has been suggested but its role is not completely clear under physiological and pathological conditions [25,26]. In addition, the association between altered ACE1 expression in tissues (brain, muscle, heart, and vessels) and the development and progression of age-related conditions such as Alzheimer disease, sarcopenia, and cardiovascular disease has been suggested, but results are controversial [17,27-30].

There are few studies showing the association between ACE1 expression in cells from the immune system (monocytes and T cells) and the progression of kidney and cardiovascular disease [8,9,31,32]. Therefore, considering the lack of information on this issue, we questioned whether ACE1 (CD143) was highly expressed in cells from the immune system during the aging process. We found that ACE1 was expressed in almost 100% of T (CD4+ and CD8+) and B lymphocytes and in all phenotypes of these cells. In nonlymphoid cells, mean ACE1 expression was 56.9% (SD 20.6%). In agreement with our findings, independent studies showed that T cells from healthy donors and monocytes from patients with congestive heart failure expressed ACE1, but there has been no investigation on cell phenotypes [25,26]. Our study is the first to show that either inexperienced (naïve) or fully activated (memory) cells expresses ACE1. Our findings suggest that the expression of ACE1 in lymphoid and nonlymphoid cells reflects health status, since our studied population presented changes in physiological parameters and high levels of ACE1 expression in immune cells. Previous independent studies showed that patients with unstable angina [32] or acute myocardial infarction [33] presented higher expression of ACE1 in T cells and dendritic cells than control subjects. In addition, markers of cell (lymphoid and nonlymphoid) functional status, such as inflammatory or growth factor production, could be modulated by ACE inhibitors (ACEi). Accordingly, mononuclear leukocytes from healthy subjects incubated with an endotoxin exhibited high levels of tissue factor activity, which was reduced in the presence of captopril in a dose-dependent pattern. This result could be related to the antithrombotic effect of ACEi [34]. In patients with congestive heart failure, immune cells cultured with lipopolysaccharide secreted high levels of the proinflammatory tumor necrosis factor α, and these levels were significantly reduced in the presence of captopril [35].”

2. In those previous studies, ACE2 has also has been reported; why is it not studied here?

Answer: Our subsequent studies will be focused on ACE1 and ACE2 expression in cells from the immune system in both younger and older adults.

3. In the limitations paragraph, the authors fairly mention the real problem—the small sample size, and I would like to add a lack of younger subjects.

Answer: We agree with the limitations pointed, and the text was changed as required:

“This study has limitations such as the small sample size and the lack of young adults for comparison. As an example, the subject presenting the highest CRP and albumin levels also exhibited a high percentage of ACE1 expression in T cells (CD4+ and CD8+), B cells, and nonlymphoid cells, in addition to the lowest percentage of CD4+ naïve cells, and the highest percentage of CD8+ terminally differentiated (EMRA) and DN B cells. However, due to the small sample size, it was not possible to associate the high expression of ACE1 in immune cells with inflammaging and immunosenescence. Correlation of physiological parameters and health status with ACE1 expression and investigating whether age and associated chronic diseases could lead to increased ACE1 expression would yield important information.”

4. The point regarding the COVID-19 pandemic, seemingly worth mentioning, is too far from this study and unnecessary.

Answer: Our point was to emphasize the negative impact of chronic diseases for the outcome of the aging population during a viral infection and how ACE1/ACE2 expression could provide information regarding diagnosis and treatment. Therefore, we would like to maintain this information.

5. Linguistic checking would improve the manuscript.

Answer: We checked for possible language errors.

### Reviewer Calogero Caruso [7]

Dear reviewer, thank you very much for suggesting revisions to our manuscript. It is a privilege to have a manuscript reviewed by a researcher with high expertise on the field of ageing. Please find our responses to your questions and corresponding revisions made to the main text.

#### General Comments

1. The paper is essentially anecdotal because it studies the cells of 6 subjects without any comparison with other age groups. There is also a serious limitation because beyond the age and sex, there is no information on the donors (how and why they were recruited, what drugs they took, etc).

Answer: It is really a limitation to have only 6 individuals for the study, but they were the only ones meeting the criteria of the proposed study. The samples were from a central bank of cells at the UNIFESP, and participants were considered “healthy” but there was no further information in addition to what we displayed in the tables in the manuscript. They were not living on homecare or hospitalized.

Our aim was to evaluate samples from individuals aged 60-69 years before the COVID-19 pandemic or vaccination. In addition, there were no samples maintained under the same conditions (PBMCs at –80 °C), obtained from young individuals, and using fresh blood could yield a result that could not be compared mainly for myeloid cells and B cells as shown in Braudeau et al [8]. Our goal from now on is to expand this study with young and older adults’ samples, since it is important to understand whether ageing is associated with an increase in ACE expression in immune cells.

2. To infer that chronological and biological ages do not match is inappropriate in the absence of the above information.

Answer: This information regarding chronological and biological age was required by another reviewer. I agree that the concept does not match without more information on the donors. However, the information is now provided in Vasto et al [9] and should be considered when older adults are studied.

3. However, the paper is of some interest because there are few studies on the topic.

Answer: Thank you for this positive comment. Few studies on the topic are the reason why we decided to send the manuscript for publication, even though there some important information on the donors is missing and a limited number of individuals was included.

#### Specific Comments

Essential revisions that are required to verify the manuscript

1. Although we do not have data on donors, placing an age and gender column in all tables adds a minimum of useful information for the reader.

Answer: The first table was submitted with age, but per requirement of MedRxiv, gender and age could not be linked to the metabolic results to preserve the anonymity of the donors.

2. Inflammaging means low grade of inflammation. The CR*P* value of 23.1 suggests acute inflammation (also because albumin has high values, while in chronic inflammation its values decrease). Therefore the averages do not have to take this subject into account.

Answer: Thank you for this comment. In a review of the literature, Heumann et al [10] found a CRP variation from 0.1 to 19.8. There is also an article from your group [11] showing that a CRP level of <5 g/dL and >5 g/dL will be considered to investigate how ageing impacts CRP levels. Considering the already small number of donors, data were maintained and statistics (mean and SD) with and without 23.1 mg/dL are now shown.

This will be the new version (Discussion) with respect to CRP: “Carlsson’s [22] study found that the CRP level was 2.6% with a coefficient variation of 1.4%, whereas in our study, we observed higher levels of CRP in 5 out of 6 individuals. Increased CRP levels have been associated with inflammaging, and our findings show that the study population has changes in functional parameters, which are likely associated with an inflammatory profile [24].”

However, an individual presented CPR 23.1 mg/dL, suggesting acute inflammation instead, but as all donors were not hospitalized or living on homecare, this sample was considered a part of the study. Another study [12] evaluating gait speed found CRP levels varying from 0.1 to 19.8 mg/dL. Our study has an important limitation, that is, the lack of data on donors such as the use of continuous medicaments or sarcopenia, hypertension, and cognition, among others, and thus it was not possible to correlate CRP with age-related conditions.

Table 1. Updated

Other suggestions to improve the manuscript

1. The authors write that their findings suggest that ACE1 could play a role in several processes linked to aging including the generation and activation of autoimmune cells, due to the experimental evidence that inhibitors of ACE suppress the autoimmune process in a number of autoimmune diseases such as EAE, arthritis, autoimmune myocarditis [13]. They do not appear to have these findings in their paper. So, they need to change the sentence.

Answer: The sentence has been changed as follows: “Our findings suggest that ACE1 could play a role in several processes linked to aging, including the generation and activation of autoimmune cells, due to the experimental evidence that inhibitors of ACE1 suppress the autoimmune process in a number of autoimmune diseases such as experimental autoimmune encephalomyelitis, arthritis, autoimmune myocarditis [49].”
